# 诱导化疗无效的局限期小细胞肺癌可能不宜原方案同期放化疗

**DOI:** 10.3779/j.issn.1009-3419.2016.12.11

**Published:** 2016-12-20

**Authors:** 大权 王, 利明 徐, 路军 赵, 文成 章, 青松 庞, 宁波 刘, 曦 陈, 秀丽 陈, 智勇 袁, 平 王

**Affiliations:** 300060 天津，天津医科大学肿瘤医院放疗科，国家肿瘤临床医学研究中心，天津市肿瘤防治重点实验室 Department of Radiotherapy, Tianjin Medical University Cancer Institute and Hospital, National Clinical Research Center for Cancer, Key Laboratory of Cancer Prevention and Therapy, Tianjin 300060, China

**Keywords:** 肺肿瘤, 诱导化疗, 同期放化疗, 二线方案, 预后, Lung neoplasms, Induction chemotherapy, Concurrent chemoradiotherapy, Second-line regimens, Prognosis

## Abstract

**背景与目的:**

小细胞肺癌(small cell lung cancer, SCLC)具有高度化疗敏感性，耐药患者不足15%，本研究拟通过回顾性分析诱导化疗无效的局限期SCLC患者放化疗次序和放疗时机与无进展生存期(progression-free survival, PFS)及总体生存(overall survival, OS)的相关性，以探索同期放化疗是否优于序贯放化疗。

**方法:**

收集2009年1月-2014年12月初治的67例诱导化疗无效的局限期SCLC，分为同期放化疗组32例与序贯放化疗组35例。94%患者临床分期为Ⅲ期，6%患者为Ⅰb期-Ⅱb期。25例行脑预防性照射(prophylactic cranial irradiation, PCI)。*Kaplan-Meier*法计算生存率并*Log-rank*法检验，组间分类数据行卡方检验。

**结果:**

全组2年OS、PFS及局部控制(local control rate, LCR)分别为53.7%、20.9%和58.2%。同期放化疗组与序贯放化疗组2年OS分别为37.5%和54.3%(*P*=0.048)、2年PFS分别为12.5%与28.6%(*P*=0.149)。同期放化疗组中，13例患者(40.6%)同步化疗方案改为二线化疗方案，19例患者仍为EP或EC方案，二者2年OS分别为53.8%与26.3%(*P*=0.741)。同期放化疗组血液学毒性反应多于序贯放化疗组(*P*=0.031)、3级放射性食管炎、放射性肺炎及胃肠道反应有增多的趋势(9.4% *vs* 0, *P*=0.176; 12.5% *vs* 2.9%, *P*=0.318; 12.5% *vs* 2.9%, *P*=0.109)。PCI与否2年OS分别为56.0%和38.1%(*P*=0.029)、PFS分别为24%和19%(*P*=0.012)。

**结论:**

诱导化疗无效的局限期SCLC可能不宜继续原方案同期放化疗，可以换用二线方案或者进行单纯放疗，由于是回顾性小样本研究，此结论还需进一步的大样本前瞻性研究证实。

## Abstract (en)

**Background and objective:**

The group of small cell lung cancer (SCLC) are usually highly sensitive to chemotherapy, and less than 15% of them are resistant to drugs. We respectively evaluate the correlation of the sequence and timing of radiotherapy with progression-free survival (PFS) and overall survival (OS) in patients with limited-stage SCLC (LS-SCLC), and to figure out whether concurrent chemoradiotherapy is superior to sequent chemoradiotherapy.

**Methods:**

Sixty-seven patients diagnosed with LS-SCLC from January 2009 to June 2014 failed to respond to induction chemotherapy. According to the sequence of therapy, they were divided into concurrent chemoradiotherapy group (*n*=32) and sequent chemoradiotherapy group (*n*=35). Ninety-four percent of the patients were diagnosed with stage Ⅲ, and six percent were stage Ib-IIb. Twenty-five patients were treated with prophylactic cranial irradiation (PCI). The *Kaplan-Meier* method was used to calculate survival time and *Log-rank* test was used for between-group comparisons. Between-group comparison of categorical data was made by *χ*^2^ test.

**Results:**

In all patients, the 2-year OS, PFS and LC rates were 53.7%, 20.9% and 58.2%. The 2-year OS and PFS rates of concurrent chemoradiotherapy group and sequent chemoradiotherapy were 37.5% *vs* 54.3% (*P*=0.048) and 12.5% *vs* 28.6% (*P*=0.149). Hematologic toxicities were more common in concurrent group than sequent one (*P*=0.031), and no statistical difference was observed between the two groups in terms of grade 3 radiation esophagitis, pneumonitis and gastrointestinal reactions (9.4% *vs* 0, *P*=0.176; 12.5% *vs* 2.9%, *P*=0.318; 12.5% *vs* 2.9%, *P*=0.109). Patients treated with PCI have superior OS and PFS comparing with those not (56.0% *vs* 38.1%, *P*=0.029; 24% *vs* 19%, *P*=0.012).

**Conclusions:**

Concurrent chemoradiotherapy with original chemotherapy regimens may not be suitable for patients who failed to respond to induction chemotherapy in LS-SCLC, and second-line regimens or radiotherapy alone can be used for them, but prospective trils with large sample are still needed to confirm that.

同期放化疗是局限期小细胞肺癌(small cell lung cancer, SCLC)的标准治疗方法^[1]^。由于SCLC具有较高的放化疗敏感性，诱导化疗有效率可达67%-80%^[2-5]^，故对于体质较好的患者，临床上常采用诱导化疗后再予以同期放化疗的治疗方法。多项研究^[6, 7]^显示诱导化疗疗效是影响局限期SCLC的预后因素，诱导化疗无效的患者与有效的相比总体生存(overall survival, OS)及无进展生存期(progression-free survival, PFS)均较差。如何改善诱导化疗无效患者的预后应当成为我们关注的重点。本研究回顾性分析67例诱导化疗无效的局限期SCLC患者的临床资料，比较不同治疗方式及放疗时机与预后的关系，从而为分层治疗提供依据。

## 1 材料与方法

### 1.1 纳入标准

① 所有患者经病理学或细胞学诊断为SCLC；②结合临床表现及辅助检查结果，确定病变为局限期；③接受过诱导化疗，根据影像学检查结果，确定为诱导化疗无效；④诱导化疗后接受胸部根治性放疗；⑤不合并第二原发癌或其他严重疾病。

### 1.2 一般临床资料

2009年1月-2014年12月在我院初治的67例局限期SCLC患者纳入本研究，其中男性51例，女性16例，年龄35岁-75岁(中位数57岁)，详细资料见表 1。

**1:** 患者一般临床资料 Clinical data of the patients

Variable	*n* (%)
Smoking index (≥400)	45 (67.2)
Loss of weight (≥5%)	8 (11.9)
KPS≥80	64 (95.5)
Gender (male)	51 (76.1)
T stage	
T_1_	5 (7.5)
T_2_	35 (52.2)
T_3_	17 (25.4)
T_4_	10 (14.9)
N stage	
N_0_	3 (4.5)
N_1_	5 (7.5)
N_2_	40 (59.7)
N_3_	19 (28.4)
Clinical stage	
Ⅰb	2 (3.0)
Ⅱb	2 (3.0)
Ⅲa	38 (56.7)
Ⅲb	25 (37.3)
PCI	25 (37.3)
KPS: Karnofsky performance status; PCI: prophylactic cranial irradiation.	

### 1.3 治疗方法

① 化疗：患者诱导化疗周期为1-6周期(中位数2周期)；32例患者(47.8%)接受同期放化疗，化疗周期为1-2周期(中位数2周期)。一线化疗方案包括依托泊苷(100 mg/m^2^，d1-d3或100 mg，d1-d5)加顺铂(60 mg/m^2^-75 mg/m^2^，d1或40 mg，d1-d3)或卡铂(300 mg/m^2^, d1)，二线化疗方案包括铂类(顺铂/卡铂/奈达铂)为基础的双药联合方案或单药化疗，化疗药物包括拓扑替康(1.4 mg/m^2^, d1-d5)、伊立替康(60 mg/m^2^, d1, d8, d15)、紫杉醇(135 mg/m^2^-175 mg/m^2^, d1)、多西他赛(75 mg/m^2^, d1)。②胸部放疗：所有患者均采用三维适形放疗(three dimensional conformal radiotherapy, 3D-CRT)或调强放射治疗(intensity modulated radiation therapy, IMRT)技术。肿瘤区(gross tumor volume, GTV)包括影像学可见的肿瘤病灶与阳性淋巴结，计划肿瘤区(plan gross tumor volume, PGTV)为GTV三维方向上均匀外扩5 mm，临床靶区(clinical tumor volume, CTV)在GTV基础上均匀外放5 mm，并包括诱导化疗前影像学证实受累淋巴引流区，计划靶区(plan tumor volume, PTV)在CTV基础上上下外扩5 mm，左右、前后方向各外扩5 mm(部分患者放疗开始后每周一次CBCT验证)。处方剂量PTV为50 Gy-60 Gy/25 f-30 f。20例患者(29.9%)行同步减量放疗，处方剂量为：PTV 54 Gy/30 f，PGTV 60 Gy/30 f。正常器官限量：脊髓最大剂量 < 45 Gy；肺V20 < 30%(同步化疗肺V20 < 28%)，平均肺剂量 < 16 Gy；食管V50 < 50%，Dmax < 64 Gy；心脏V30 < 40%。1-2周期诱导化疗后引入放疗定义为早放疗，3-6周期诱导化疗后引入放疗定义为晚放疗。

### 1.4 疗效及不良反应评价

诱导化疗疗效评价在化疗结束后1周-2周内进行，评价基线为诱导化疗前CT所示病灶大小；放疗后疗效评价在放疗后3个月内进行，评价基线为放疗前CT所示病灶大小。根据实体瘤疗效评价标准(Response Evaluation Criteria in Solid Tumors, RECIST)1.1^[8]^，可分为完全缓解(complete regression, CR)、部分缓解(partinal regression, PR)、病情稳定(stable disease, SD)、病情进展(progressive disease, PD)；有效为CR+PR，无效为SD+PD(多周期诱导化疗的患者化疗期间存在多次疗效评价，最后一次疗效评价结果为SD/PD者纳入为诱导化疗无效)。急性不良反应根据美国国家癌症研究所常见不良反应事件评价标准(National Cancer Institute Common Terminology Criteria for Adverse Events, NCI CTC AE)3.0标准^[9]^进行评价，主要包括血液学不良反应、消化道不良反应、放射性食管炎及放射性肺炎。

### 1.5 观察终点

本研究观察终点包括局部控制(local control rate, LCR)、OS和PFS。局部失败定义为从治疗开始到原发病灶处(纵隔区域和/或颈部淋巴引流区)肿瘤进展或复发。OS定义为从治疗开始时间到病例因任何原因导致的死亡时间、截尾时间或末次随访时间。PFS定义为治疗开始时间至疾病进展/复发时间或病例因任何原因导致的死亡时间或末次随访时间。末次随访时间为2015年9月10日。

### 1.6 统计学方法

采用SPSS 17.0统计学软件进行统计学分析，*Kaplan-Meier*法分析OS及PFS并进行*Log-rank*法检验，分类数据组间比较进行卡方检验。*P* < 0.05为差异有统计学意义。

## 2 结果

### 2.1 全组疗效

诱导化疗后疗效为SD、PD分别为44例(65.7%)、23例(34.3%)；30例晚放疗患者中(诱导化疗周期为3-6)，8例患者为获得性耐药(首次化疗后疗效评价为PR，末次为SD/PD)，17例患者为原发耐药(首次化疗后疗效评价即为SD/PD)，5例患者仅在全部诱导化疗结束后进行疗效评价，其耐药类型无法确定。放疗后近期疗效为CR、PR、SD、PD分别为1例(1.5%)、40例(59.7%)、12例(17.9%)、14例(20.9%)。截至2015年9月10日随访时间3.68个月-54.54个月(中位随访时间16.03个月)，1年、2年、3年OS及中位OS分别为79.1%、53.7%、35.8%和18.69个月，PFS分别为53.7%、20.9%、17.9%和12.40个月，LC分别为80.6%、58.2%、52.2%和19.30个月。

### 2.2 单因素分析

单因素分析结果显示，吸烟史(*P*=0.025)、同期放化疗(*P*=0.048)、PCI(*P*=0.029)是影响OS的因素；性别(*P*=0.287)、体重下降(*P*=0.750)、T分期(*P*=0.996)、N分期(*P*=0.732)、肿瘤-淋巴结-转移(tumor-node-metastasis, TNM)分期(*P*=0.654)、放疗时机(*P*=0.468)不是影响OS的因素。吸烟史(*P*=0.028)、PCI(*P*=0.029)是影响PFS的因素；性别(*P*=0.997)、体重下降(*P*=0.585)、T分期(*P*=0.303)、N分期(*P*=0.261)、TNM分期(*P*=0.528)、放疗时机(*P*=0.063)、同期放化疗(*P*=0.149)不是影响PFS的因素。

### 2.3 多因素分析

将单因素分析中*P* < 0.1的因素(包括吸烟史、放疗时机、同期放化疗、PCI)纳入多因素分析，结果显示，吸烟史(*P*=0.034, HR=1.917, 95%CI: 1.049-3.504)、放疗时机(*P*=0.012, HR=2.119, 95%CI: 1.182-3.798)、PCI(*P*=0.007, HR=0.444, 95%CI: 0.246-0.804)是影响PFS的因素；吸烟史(*P*=0.033, HR=2.108, 95%CI: 1.060-4.191)、PCI(*P*=0.039, HR=0.510, 95%CI: 0.269-0.968)是影响OS的因素。

### 2.4 不同放疗时机分组疗效比较

不同放疗时机的一般临床资料具有可比性，详见表 2。早放疗与晚放疗组患者中位生存时间分别为18.17个月(12.573-23.764)与19.55个月(11.642-27.454)，2年OS分别为40.5%与56.7%(*P*=0.468)、中位PFS分别为10.19个月(8.583-11.787)与13.34个月(11.796-14.882)，2年PFS分别为16.2%与26.7%(*P*=0.063)。进一步分析早放疗组同期放化疗(21例)与序贯放化疗(16例)的2年OS分别为33.3%与50%(*P*=0.180)、PFS分别为9.5%与25%(*P*=0.511)；晚放疗组同期放化疗(11例)与序贯放化疗(19例)的2年OS分别为36.4%与57.9%(*P*=0.291)、PFS分别为18.2%与31.6%(*P*=0.433)。

**2:** 不同放疗时机分组的一般临床资料 Clinacal data of groups according to the timing of radiotherapy

Variable	Early radiotherapy [*n* (%)]	Late radiotherapy [*n* (%)]	*P*
Median age (year)	59	57	0.743
Gender (male)	28 (75.7)	23 (76.7)	0.392
Smoking index (≥400)	25 (67.6)	20 (66.7)	0.938
Loss of weight ( > 5%)	5 (13.5)	3 (10.0)	0.659
KPS≥80	35 (91.9)	29 (96.7)	0.683
T stage			0.458
T_1_	4 (10.8)	1 (3.3)	
T_2_	17 (45.9)	18 (60.0)	
T_3_	11 (29.7)	6 (20.0)	
T_4_	5 (13.5)	5 (16.7)	
N stage			0.746
N_0_	2 (5.4)	1 (3.3)	
N_1_	2 (5.4)	3 (10.0)	
N_2_	21 (56.8)	19 (63.3)	
N_3_	12 (32.4)	7 (23.3)	
Clinical stage			0.514
Ⅰb	2 (5.4)	0	
Ⅱb	1 (2.7)	1 (3.3)	
Ⅲa	19 (51.4)	19 (63.3)	
Ⅲb	15 (40.5)	10 (33.3)	
PCI	14 (37.8)	11 (36.7)	0.921
Therapy			0.102
Concurrent group	21 (56.8)	11 (36.7)	
Sequent group	16 (43.2)	19 (63.3)	

### 2.5 同期放化疗与序贯放化疗分组疗效比较

一般资料具有可比性，详见表 3。同期放化疗组与序贯放化疗组患者中位生存时间分别为17.15个月(11.69-22.61)与24.28个月(5.181-43.377)，2年OS分别为37.5%和54.3%(图 1A，*P*=0.048)；中位PFS分别为11.14个月(7.292-14.983)与12.88个月(11.378-14.379)，2年PFS分别为12.5%与28.6%(图 1B，*P*=0.149)。另外，在同期放化疗组中，13例患者同步化疗方案改为二线化疗方案，19例患者仍为EP或EC方案，二者2年OS分别为53.8%与26.3%(*P*=0.741)、PFS分别为15.3%与10.5%(*P*=0.800)。在序贯放化疗组中，10例患者在放疗后引入了二线化疗方案，25例患者仍为EP或EC方案，二者2年OS分别为60%与56%(*P*=0.606)，2年PFS分别为20%与32%(*P*=0.668)。

**3:** 同期放化疗组与序贯放化疗组的一般临床资料 Clinical data of groups according to the sequence of therapy

Variables	Concurrent group [*n* (%)]	Sequent group [*n* (%)]	*P*
Median age (year)	57	60	0.419
Gender (male)	27 (84.3)	24 (68.6)	0.130
Smoking index (≥400)	24 (75.0)	21 (60.0)	0.192
Loss of weight (≥5%)	4 (10.8)	4 (13.3)	0.893
KPS≥80	31 (96.9)	33 (94.3)	0.609
T stage			
T_1_	4 (6.3)	1 (8.6)	0.119
T_2_	13 (43.8)	22 (57.1)	
T_3_	11 (34.3)	6 (17.1)	
T_4_	4 (12.5)	6 (17.1)	
N stage			
N_0_	0	3 (8.6)	0.358
N_1_	2 (6.3)	3 (8.6)	
N_2_	21 (65.6)	19 (59.4)	
N_3_	9 (28.1)	10 (28.6)	
Clinical stage			
Ⅰb	0	2 (5.7)	0.271
Ⅱb	0	2 (5.7)	
Ⅲa	19 (59.4)	19 (54.3)	
Ⅲb	13 (40.6)	12 (34.3)	
Timing of radiotherapy			0.102
Early radiotherapy	21 (65.6)	16 (45.7)	
Late radiotherapy	11 (34.4)	19 (54.3)	
PCI	14 (43.8)	11 (31.4)	0.298

**1:**
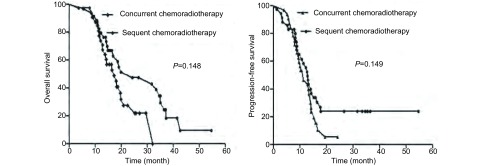
序贯放化疗组与同期放化疗组OS曲线(A)及PFS曲线(B) Kaplan-Meier analysis of overall survival (OS)(A) and progression-free survival (PFS)(B) according to the sequence of chemoradiotherapy

### 2.6 PCI与否疗效比较

全组患者接受PCI者25例(37.3%)，其中PCI组与无PCI组2年OS分别为56.0%和38.1%(*P*=0.029)、PFS分别为24%和19%(*P*=0.012)。

### 2.7 放疗急性毒性反应

详见表 4。

**4:** 同期放化疗组和序贯放化疗组的放疗急性毒性反应 The acute side effects of patients in concurrent and sequent group

Variables	Concurrent group [*n* (%)]	Sequent group [*n* (%)]	*P*
Hematologic toxicity			0.031
Grade 1-2	14 (43.8)	8 (22.9)	
Grade 3-4	4 (12.5)	1 (2.9)	
Radiation pneumonitis			0.318
Grade 2	2 (6.3)	2 (6.3)	
Grade 3	4 (12.5)	1 (2.9)	
Radiation esophagitis			0.176
Grade 2	2 (6.3)	2 (5.7)	
Grade 3	3 (9.4)	0	
Gastrointestinal effects			0.109
Grade 2	5 (15.6)	2 (5.7)	
Grade 3	4 (12.5)	1 (2.9)	

### 2.8 失败分析

全组共53例患者出现了治疗失败，其中局部复发16例，远处转移24例，局部+远处进展13例。同步放化疗组28例治疗失败，其中局部复发7例、远处转移13例、局部+远处进展8例；序贯放化疗组25例治疗失败，其中局部复发11例、远处转移9例、局部+远处进展5例。远处转移失败患者中同期放化疗组21例、序贯放化疗组14例(*P*=0.036)。

## 3 讨论

Reymen等^[10]^研究发现，GTV总体积(包括转移淋巴结)是影响Ⅰ期-Ⅲ期SCLC总体生存的独立预后因素。局限期SCLC的标准治疗方法是同期放化疗^[11]^，诱导化疗的加入，使患者肿瘤负荷降低，减小了GTV体积及正常肺、心脏及食管受量，降低了放疗毒副反应，进而使患者生存获益^[6, 12]^。相关研究^[3, 7, 13]^显示，局限期SCLC患者诱导化疗(2-6周期)后再行放化疗，中位OS为20个月-25.4个月，中位PFS为12个月-15.4个月。而在本研究中，全组患者中位PFS为12.40个月，中位OS仅为18.69个月，这是因为本研究纳入病例全部为诱导化疗无效患者，而上述研究中诱导化疗无效的患者仅占21.9%-34%。在Fujii等^[6]^的研究中，全组均为诱导化疗有效的患者，其中位OS达39.6个月。由于存在化疗抗拒，GTV体积经诱导化疗后无明显缩小，故诱导化疗无效的患者在后期放化疗次序及放疗引入时机的优化选择上，可能异于诱导化疗有效的患者。有研究^[7]^指出，早放疗能提高诱导化疗有效患者的PFS与OS，而对诱导化疗无效的患者却无影响。另外，在放化疗次序方面，对于诱导化疗无效的患者，后期行同期放化疗是否优于序贯放化疗，同期化疗方案是否要尽早改为二线方案，目前尚无大样本比较。

多项临床研究^[14, 15]^显示，尽早引入放疗能提高局限期SCLC的PFS及OS。根据临床指南，放疗应在化疗的第1-2周期引入，推荐类别为Ⅰ类^[16]^。由于SCLC对化疗具有高度敏感性，原发耐药患者不足15%，故上述研究涉及的病例也多为诱导化疗有效的患者，因而其结论并不完全适用于原发耐药的患者。目前在放疗时机的选择上仍有争论，近期Lu等^[17]^的*meta*分析显示，对于肿瘤体积较大或老年患者，晚放疗优于早放疗。在本研究中，虽然多因素分析显示，放疗时机是影响PFS的独立预后因素，但是早放疗组和晚放疗组的OS差异无统计学意义。化疗耐药的患者经诱导化疗后肿瘤体积无明显缩小，由于GTV体积较大，较早引入放疗可能无法使患者明显获益。因此，仍需设计前瞻性临床试验，研究放疗时机对耐药患者远期疗效的影响。

虽然同期放化疗已成为SCLC的标准治疗方法，但是诱导化疗耐药的患者继续原方案同期化疗能否使患者获益，是否要尽早改为二线方案，目前尚无定论。本项研究发现，序贯放化疗组的OS反而优于同期放化疗组(*P*=0.048)，二者PFS差异无统计学意义(*P*=0.149)，多因素分析显示同期放化疗不是影响OS的独立因素。该结果可以从两个方面进行解释，一方面，同期放化疗组患者在诱导化疗无效后只有13例(40.6%)患者改用二线方案，化疗耐药的患者由于肿瘤细胞存在化疗抗性，放疗过程中继续进行原方案化疗可能无法有效发挥其放疗增敏及对肿瘤细胞进行独立杀伤的作用。虽然亚组分析显示同期化疗组中，更换二线方案与未更换的患者相比，OS差异无统计学意义(*P*=0.741)，但是这可能是由于病例数较少所致，而且在数值上，换用二线化疗方案后OS显示出一定优势(53.8% *vs* 26.3%)。另外，换用二线化疗方案的患者中，9例患者(69.2%)使用紫杉醇/多西他赛。Tiseo等^[18]^指出对于一线化疗耐药的患者，部分二线药物如紫杉醇有效率可达20%-29%。同期放化疗过程中改用二线方案可能优于继续原方案化疗。另一方面，考虑诱导化疗的主要目的在于缩小后期放疗靶区范围，降低正常组织受量，提高患者放化疗耐受能力，而化疗耐药的患者由于肿瘤负荷无明显变化，靶区范围较大，正常组织受量也会相应提高，再行同期放化疗毒副反应必然增加；本研究结果显示，同期放化疗组的血液学毒性反应多于序贯放化疗(*P*=0.031)，而3级放射性肺炎、放射性食管炎及胃肠道反应比例也多于序贯放化疗组(12.5%、9.4%、12.5% *vs* 2.9%、0、2.9%)。因此，化疗耐药的SCLC患者可能不宜继续应用原方案同期放化疗，可以换用二线方案的同期放化疗，或者为了减轻毒性反应而进行单纯放疗。

脑是SCLC常见转移部位，AuPerin等^[19]^的*meta*分析显示对于初始治疗有效的病例行PCI能够延长PFS及OS。但是近期日本的Ⅲ期临床研究显示，广泛期SCLC初始治疗有效后行PCI不能延长生存，这促使2014版日本肺癌学会肺癌治疗指南将广泛期SCLC行PCI的推荐级别由A降为B^[20]^。但是对于局限期SCLC，初始治疗后CR/PR患者行PCI，指南上依然是A类推荐^[20]^。在本研究中，多因素分析显示，脑预防照射是影响OS及PFS的良好预后因素，这与既往研究^[19, 21]^相一致。诱导化疗无效的患者由于存在化疗抗拒，可能后期更需引入PCI以降低脑转移风险，因此，对于诱导化疗无效的患者经放化疗后可以行PCI。

值得注意的是，在本研究中，54例患者为原发耐药(首次化疗后疗效评价即为SD/PD)，8例患者为获得性耐药(可能由于化疗周期过多而耐药)，不同耐药类型是否也需要分层治疗，仍需进一步研究。另外，受限于回顾性研究的性质，本研究在病例选择存在不足之处，例如9例患者放疗前接受5-6周期化疗，已经不属于诱导化疗的范畴，但由于其所占比重较小，故也纳入本项研究。

综上所述，当前的SCLC治疗指南可能并不完全适用于一线化疗耐药的SCLC患者，对于诱导化疗无效的患者，1-2周期即引入放疗可能并不能使其明显获益，诱导化疗无效后继续原方案同期放化疗无法使患者生存获优势。本研究受限于回顾性研究的性质，且病例数有限，只能提示初步的倾向性，无法得出准确的结论。但是一线化疗耐药的SCLC患者应该行个体化治疗，尚需开展大规模前瞻性临床试验进一步研究。
